# Character-based identification system of scombrids from Indian waters for authentication and conservation purposes

**DOI:** 10.1080/23802359.2020.1810144

**Published:** 2020-08-26

**Authors:** Sonalismita Mahapatra, Rathipriya A., Priyadarshini Dwivedy, Suresh E., Shanmugam S. A., Kathivelpandian A.

**Affiliations:** Institute of Fisheries Postgraduate Studies, TNJFU OMR Campus, Vaniyanchavadi, Chennai

**Keywords:** Scombrids, CBIS, species-specific probes, BLOG 2.0, authentication

## Abstract

Scombrids are the important component of pelagic fishery resources which include 54 species under 15 genera commonly known as mackerels, bonitos, and tunas. Due to the high commercial value attained, there are real chances of fraudulent substitution by species of inferior value. DNA based species identification methods can be applied to detect product adulteration, as well as to better contribute to the conservation and management of these species by providing accurate species identification independently of the age of the individuals or the tissue processed. In this study, a total of 15 commercially important scombrid species from Indian waters were analyzed. Due to the inadequacy of mitochondrial COI barcoding gene in discriminating between some *Thunnus* species, cytochrome b sequences were used instead. For all the 15 species, we propose a DNA character-based keys which uses a diagnostic combination of nucleotides and respective probes, including the first character-based keys and probes to differentiate between *Thunnus albacares* and *T. obsesus*.

## Introduction

Seafood is regarded as both an important protein source and a delicacy for human populations across the globe, and is mainly obtained from pelagic fishery resources. Among pelagicfishes, scombrids, commonly known as mackerels, bonitos, and tunas are athe major component with 54 species under 15 genera. The total marine fish landings of Indian waters is 3.49 million tonnes to which the pelagic ecosystem contributes 54% (FRAD, CMFRI 2018). Out of the 21 species reported in Indian waters (Venkataraman and Sivaperuman 2014), eight species alone contribute to over 99% of the fisheries (CMFRI 2015). Scombrids contribute 10% of the total marine landings of Indian waters (FRAD, CMFRI 2018). Among scombrids, seer fishes are one of the important exploited resources in the country yielding a total 49,209 tonnes and comprising 24.8% of the total large pelagic fishery with high domestic and exports value (CMFRI 2015).

Due to the high price attained, scombrids are prone to get adulterated with other fishes (*Pangasius* species), in some restaurants and processing industries (Danni 2015). In these conditions, fish identification through the traditional methods such as morpho-meristic measurements (Strauss and Bond 1990) and otoliths (Granadeiro and Silva 2000) is difficult. Further, eggs and larvae (especially those of the genus *Thunnus*) generally cannot be distinguished easily by morphological characters (Richards et al. 1990) for the purpose of conservation and management of these important fishery resources. DNA-based identification methods have been developed to address these issues. In most cases, the mitochondrial COI gene is used for fish identification and authentication purposes (Lakra et al. 2011; Bingpeng et al. 2018). Several studies have been carried out to utilize the mitochondrial genes for species identification purposes. There were studies using molecular tools for different groups of scombrid fishes. Molecular identification of scombrids has been ascertained by Miya et al. (2013) using complete whole mitogenome analysis. Similarly, the complete mitochondrial genome sequence of *R. kanagurta* is also studied by Chen et al. (2013) for systematic studies. The differentiation of tuna products using 464 bp of *Cyt b* sequences was reported by Abdullah and Rehbein (2015a). Wherein, speed and accuracy for authentication purposes demands alternate methods than PCR sequencing.

Furthermore, the limitations of using full-length sequences which might require technical expertise, and due to lack of a standard short-barcode fragment, most of the character-based studies have targeted the full-length COI barcode (Mutanen et al. 2015), although extraction of long DNA fragment is difficult in cases of archival specimen and processed biological material (Wandeler et al. 2007). Due to the inadequacy of mitochondrial COI gene in discriminating *Thunnus* species (Puncher et al. 2015), it is needed to supplement alternative mitochondrial genes such as cytochrome b (*Cyt b*) which is used commonly for authentication purposes (Cutarelli et al. 2018). Further, it is also necessary to develop alternative methods such as character-based identification systems as effective tools for authentication and conservation purposes. Character-based identification system (CBIS) is a very useful tool in the identification of fish species using specific locations of nucleotide sequences of the species under study. The probes developed through CBIS will be of immense use for authenticity purposes to detect the substitution of low-value fishes with highly priced ones.

In this context, this study is aimed at developing species-specific nucleotide keys through character-based identification system (CBIS) which could be used in developing a DNA chip for authentication purposes of commercially important scombrid species from Indian waters.

## Materials and methods

### Data collection

A total of 1203 mitochondrial cytochrome b sequences of commercially important species of scombrids in India were collected from GenBank, namely: *Acanthocybium solandri*, *Auxis thazard*, *Rastrelliger kanagurta*, *Thunnus tonggol*, *Auxis rochei*, *Euthynnus affinis*, *Katsuwonus pelamis*, *Rastrelliger brachysoma*, *Rastrelliger faughni*, *Scomberomorus commerson*, *Scomberomorus guttatus*, *Sarda orientalis*, *Thunnus alalunga*, *Thunnus obesus*, and *Thunnus albacares* (Supplemental online material). The number of sequences per species ranged from 15 to 284. A total of 776 sequences from 15 species were used for further analysis after checking quality and authenticity.

### Data analysis

Alignment of downloaded *Cyt b* sequences was done in Bio edit ver. 7.0.5.2 (Hall 1999) using the ClustalW tool (Thompson et al. 1997). Trimming of aligned sequences was done for making the downloaded sequences in uniform length for further analysis. BLOG 2.0 (Weitschek et al. 2012) was used for character-based species classification of scombrid group of fishes. Classification of scombrids and the quality control of the designed probes was checked with MFEprimer 3.0 server (Wang et al. 2019).

## Results

The sequences downloaded from NCBI were in the length range of 308–1141bp. The resulted length after alignment and trimming of sequences was of 640 bp. BLOG analysis yielded a total of 25 diagnostic nucleotides, with two diagnostic nucleotides for nine species and a single diagnostic nucleotide for the remaining six species. A total of 25 probes (18–37 bp)were designed for 15 species, having 9 species with two probes and 6 species with a single probe (Table 1).

**Table 1.** Species-specific formula and probes of scombrid fish species identified through CBIS.

Sl. No.	Species name	Species formula	Probe	Ta
1	*Acanthocybium solandri*	pos81 = A	CCCAAATCCTCACAGGCCTATTCC	54.5 °C
2	*Auxis thazard*	pos153 = T	GCCTATTCCTTGCAATACACTACACCCC	56.4 °C
3		pos84 = C	CCCTCCAATATTTCCGCATGATGAAACT	54.8 °C
4	*Rastrelliger kanagurta*	pos177 = T	ATACACTACACTCCCGATGTTGAATCAGCA	56.6 °C
5		pos261 = T	ACGCAAATGGCGCTTCTTTCTTCTTT	56 °C
6	*Thunnus tonggol*	pos45 = T	CTAGTTGACCTTCCTACCCCCTCTAATATTT	54 °C
7	*Auxis rochei*	pos66 = G	GTCATGATAACTGCATTCGTCGGCTA	54.7 °C
8	*Euthynnus affinis*	pos102 = A	CATGATGAAACTTTGGATCACTGCTTGG	54.3 °C
9		pos108 = G	TGGATCACTGCTTGGTCTCTGCCTTA	56.8 °C
10	*Rastrelliger brachysoma*	pos28 = G	ACACTCCCGATGTTGAATCAGCAT	54.1 °C
11	*Katsuwonus pelamis*	pos177 = A	TATACCCCTGACGTAGAATCAGCCTT	54 °C
12		pos246 = C	ATCCGAAACCTCCATGCCAACG	55.2 °C
13	*Rastrelliger faughni*	pos168 = C	ATACACTACACCCCCGACGTTGA	54.8 °C
14		pos258 = T	ACGCAAACGGTGCCTCCTTC	55.7 °C
15	*Scomberomorus commerson*	pos246 = G	GCAGATTCGATGTCCGGGGTA	53.9 °C
16	*Scoberomorus guttatus*	pos246 = T	TCCGAAACCTTCACGCAAATGG	53.7 °C
17		pos258 = A	CACGCAAATGGAGCATCCTTCTTCTT	55.4 °C
18	*Sarda orientalis*	pos81 = C	CCCCTTCCAATATCTCTGCATGATGAAATTT	54.7 °C
19		pos258 = C	GCAAACGGCGCCTCCTTTTTC	55.5 °C
20	*Thunnus alalunga*	pos258 = A	TCCACGCAAACGGGGCC	55.8 °C
		pos261 = T	CTGAATGATACTTCCTATTTGCTTACGCAATTCT	54.8 °C
21	*Thunnus obesus*	pos261 = G	CGGATTAAACTCAAATGCAGATAAAATCTCATTCCA	55.1 °C
22	*Thunnus albacares*	pos81 = C	ACAAAGGCTTGGTCCTGACTTTACTGT	55.6 °C
23		pos258 = G	ATGTCTTTCTGAGGAGCTACCGTCATTA	54.5 °C

## Discussion

For commercially important group of fishes such as scombrids, it is essential to derive the method for fast and accurate identification to manage the adulteration. Scombrids possess high degree of chance of mislabeling with other cheap fish species as morphological identification is not possible in processed fish specimens. Recent studies established the utility of CBIS as an appropriate method for identification purposes. Several studies have been carried out to generate the character-based keys (Paine et al. 2007; Lowenstein et al. 2009; Bergmann et al. 2009; Puncher et al. 2015; Vargheese et al, 2019). In order to identify the mislabeling of high-value Bluefin tuna, Lowenstein et al. (2009) developed a character-based identification system using mitochondrial COI gene for diagnosing *Thunnus* species. Similarly, Paine et al. (2007) used a character-based identification system for 17 species of scombrids from the western Atlantic Ocean. Molecular identification of Atlantic blue fin tuna larvae using *Cyt b* is also studied by Puncher et al (2015). In this study, we developed 25 positions of character-based keys for identification of scombrids from Indian waters which is in similar line with Lowenstein et al. (2009) who described 40 positions for differentiating the blue fin tuna.

CBIS could be done using several software systems such as CAOS (Bergmann et al. 2009), BLOG(Weitschek et al. 2013), etc. In this study, BLOG (Weitschek et al. 2013) is used as it has several advantages over tree-based methods and similarity methods (Van Velzen et al. 2012). Vargheese et al. (2019) used BLOG 2.0 for differentiating a total of 82 species of elasmobranchs and observed 214 diagnostic nucleotides when compared to 25 positions for scombrids from Indian waters.

In the recent years, increase in trade of sea foods leads to an increase in the occurrence of mislabeling and also fish adulteration is common by some industries. In Brazilian fish markets, 80% of the surubim (*Pseudoplatystoma* spp.) products were mislabeled (Carvalho et al. 2011). Similarly, in Italian fish markets commercialized shark samples got mislabeled by some local fish species (Scarano and Rao 2014). Abdullah and Rehbein (2015b) amplified parvalbumin intron by exon-primed intron-crossing PCR for differentiating scombrids and possible substituted species such as catfish, tilapia and snapper fish species. Characterization of amplicons was done through sequencing (tunas), single-strand conformation polymorphism (SSCP) (scombrid, catfish, tilapia, and snapper species) and restriction fragment length polymorphism (RFLP) (catfish). The SSCP method differentiated catfish, tilapia, snapper, and scombrid species except tunas. Tunas of the genus *Thunnus* had an unexpected low variability of intron sequences, which prevented their differentiation by sequencing or SSCP. Lin and Hwang (2007) developed RFLP with two sets of primers to amplify 126 and 146 bp of partial mitochondrial cytochrome b gene, and five restriction enzymes, Bsp1286I, HincII, RsaI, ScaI, and MboII were determined to analyze the short length fragments to authenticate 18 canned tunas. Despite several methods used for differentiating *Thunnus* sp, still there is a need for fast, reliable, and low-cost methods to identify processed tuna and to differentiate other scombrid species for supporting sustainable fisheries and tuna trade. In this context, the species-specific probes for 15 species of scombrids obtained in this study will be of immense help to address the similar mislabeling concerns in the commercially important scombrid group of fishes especially tunas. The developed probes could be used to develop DNA chip for faster and accurate identification of scombrid fish species.

DNA chip is well established method to differentiate multiple species at a particular point of time for food and forensic applications (Lettieri 2006). A DNA chip was developed by Kochzius et al. (2008) for 11 commercially important fish species using 16S rDNA gene.

DNA chip could be of immense use wherein a large number of species-specific nucleotides could be spotted in small space for identifying target species accurately (Kim et al. 2011). The probes developed in this study will be used for curbing the mislabeling and adulteration of commercially important scombrids apart from conservation and management of these valuable resources from Indian waters.

## Supplementary Material

Supplemental Material

## Data Availability

The availability of data used in the present study is in the NCBI database (https://www.ncbi.nlm.nih.gov/nucleotide/). The nucleotide information taken from NCBI were analyzed using various molecular tools and the findings are presented in the manuscript. The details are given as supplementary Table 1.
