# Supplementary material for: Character-based identification system of scombrids from Indian waters for authentication and conservation purposes
Source: Mitochondrial DNA B Resour. 2020 Aug 26;5(3):3221–4. doi: 10.1080/23802359.2020.1810144 (PMC7783091; doi:10.1080/23802359.2020.1810144)
Supplement: Supplemental Material [file TMDN_A_1810144_SM1804.rtf]

Supplementary material:  GenBank Accession nos. of scombrids used for CBIS
SL No.	Species Name	Accession number	
1	Auxis rochei	AB119140.1	
2	Auxis rochei	AB119139.1	
3	Auxis rochei	AB119138.1	
4	Auxis rochei	AB119137.1	
5	Auxis rochei	AB119136.1	
6	Auxis rochei	AB106861.1	
7	Auxis rochei	AB106859.1	
8	Auxis rochei	AB106858.1	
9	Auxis rochei	AB106857.1	
10	Auxis rochei	AB106856.1	
11	Auxis rochei	AB106855.1	
12	Auxis rochei	AB106854.1	
13	Auxis rochei	AB106853.1	
14	Auxis rochei	AB106852.1	
15	Auxis rochei	AB106851.1	
16	Auxis rochei	AB106850.1	
17	Auxis rochei	AB106849.1	
18	Auxis rochei	AB106848.1	
19	Auxis rochei	AB106847.1	
20	Auxis rochei	AB106846.1	
21	Auxis rochei	AB106845.1	
22	Auxis rochei	AB106844.1	
23	Auxis rochei	AB106843.1	
24	Auxis rochei	AB106842.1	
25	Auxis rochei	AB106841.1	
26	Auxis rochei	AB106840.1	
27	Auxis rochei	AB106839.1	
28	Auxis rochei	AB106838.1	
29	Auxis rochei	AB106837.1	
30	Auxis rochei	AB106836.1	
31	Auxis rochei	AB106331.1	
32	Auxis rochei	AB106330.1	
33	Auxis rochei	AB106329.1	
34	Auxis rochei	AB106328.1	
35	Auxis rochei	AB106327.1	
36	Auxis rochei	AB106326.1	
37	Auxis rochei	AB106325.1	
38	Auxis rochei	AB106324.1	
39	Auxis rochei	AB106323.1	
40	Auxis rochei	AB106322.1	
41	Auxis rochei	AB106321.1	
42	Auxis rochei	AB106320.1	
43	Auxis rochei	AB106319.1	
44	Auxis rochei	AB106318.1	
45	Auxis rochei	AB106317.1	
46	Auxis rochei	AB106316.1	
47	Auxis rochei	AB106315.1	
48	Auxis rochei	AB106314.1	
49	Auxis rochei	AB106313.1	
50	Auxis rochei	AB106312.1	
51	Acanthocybium solandri	EF653645.1	
52	Acanthocybium solandri	EF653644.1	
53	Acanthocybium solandri	EF653643.1	
54	Acanthocybium solandri	EF653642.1	
55	Acanthocybium solandri	EF653641.1	
56	Acanthocybium solandri	EF653640.1	
57	Acanthocybium solandri	EF653639.1	
58	Acanthocybium solandri	EF653638.1	
59	Acanthocybium solandri	EF653637.1	
60	Acanthocybium solandri	EF653636.1	
61	Acanthocybium solandri	EF653635.1	
62	Acanthocybium solandri	EF653634.1	
63	Acanthocybium solandri	EF653633.1	
64	Acanthocybium solandri	EF653632.1	
65	Acanthocybium solandri	EF653631.1	
66	Acanthocybium solandri	EF653630.1	
67	Acanthocybium solandri	EF653629.1	
68	Acanthocybium solandri	EF653628.1	
69	Acanthocybium solandri	EF653627.1	
70	Acanthocybium solandri	EF653626.1	
71	Acanthocybium solandri	EF653625.1	
72	Acanthocybium solandri	EF653624.1	
73	Acanthocybium solandri	EF653623.1	
74	Acanthocybium solandri	EF653622.1	
75	Acanthocybium solandri	EF653621.1	
76	Acanthocybium solandri	EF653620.1	
77	Acanthocybium solandri	EF653619.1	
78	Acanthocybium solandri	EF653618.1	
79	Acanthocybium solandri	EF653617.1	
80	Acanthocybium solandri	EF653616.1	
81	Acanthocybium solandri	EF653615.1	
82	Acanthocybium solandri	EF653614.1	
83	Acanthocybium solandri	EF653613.1	
84	Acanthocybium solandri	EF653612.1	
85	Acanthocybium solandri	EF653611.1	
86	Acanthocybium solandri	EF653610.1	
87	Acanthocybium solandri	EF653609.1	
88	Acanthocybium solandri	EF653608.1	
89	Acanthocybium solandri	EF653607.1	
90	Acanthocybium solandri	EF653606.1	
91	Acanthocybium solandri	EF653605.1	
92	Acanthocybium solandri	EF653604.1	
93	Acanthocybium solandri	EF653603.1	
94	Acanthocybium solandri	EF653602.1	
95	Acanthocybium solandri	EF653601.1	
96	Acanthocybium solandri	EF653600.1	
97	Acanthocybium solandri	EF653599.1	
98	Acanthocybium solandri	EF653598.1	
99	Acanthocybium solandri	EF653597.1	
100	Acanthocybium solandri	EF653596.1	
101	Acanthocybium solandri	EF653595.1	
102	Acanthocybium solandri	EF653594.1	
103	Acanthocybium solandri	EF653593.1	
104	Acanthocybium solandri	EF653592.1	
105	Acanthocybium solandri	EF653591.1	
106	Acanthocybium solandri	EF653590.1	
107	Acanthocybium solandri	EF653589.1	
108	Acanthocybium solandri	EF653588.1	
109	Acanthocybium solandri	EF653587.1	
110	Auxis_thazard	AY955288.1	
111	Auxis_thazard	DQ080313.1	
112	Auxis_thazard	EF141173.1	
113	Auxis_thazard	DQ497824.1	
114	Auxis_thazard	DQ497823.1	
115	Auxis_thazard	DQ497822.1	
116	Auxis_thazard	DQ497821.1	
117	Auxis_thazard	DQ497820.1	
118	Auxis_thazard	DQ497819.1	
119	Auxis_thazard	DQ497818.1	
120	Euthynnus_affinis	AY390598.1	
121	Euthynnus_affinis	DQ497827.1	
122	Euthynnus_affinis	KM055412.1	
123	Euthynnus_affinis	EU349374.1	
124	Euthynnus_affinis	EU349373.1	
125	Euthynnus_affinis	EU349372.1	
126	Euthynnus_affinis	EU349371.1	
127	Euthynnus_affinis	AB098092.1	
128	Euthynnus_affinis	AB098091.1	
129	Euthynnus_affinis	EU708972.1	
130	Euthynnus_affinis	EF141174.1	
131	Euthynnus_affinis	DQ497828.1	
132	Euthynnus_affinis	DQ497826.1	
133	Katsuwonus pelamis	DQ197958.1	
134	Katsuwonus pelamis	KJ617389.1	
135	Katsuwonus pelamis	KJ617388.1	
136	Katsuwonus pelamis	KJ617387.1	
137	Katsuwonus pelamis	KJ617376.1	
138	Katsuwonus pelamis	KJ617375.1	
139	Katsuwonus pelamis	KJ617374.1	
140	Katsuwonus pelamis	KJ617349.1	
141	Katsuwonus pelamis	KJ617345.1	
142	Katsuwonus pelamis	KJ617339.1	
143	Katsuwonus pelamis	KJ617338.1	
144	Katsuwonus pelamis	KJ617334.1	
145	Katsuwonus pelamis	KJ617333.1	
146	Katsuwonus pelamis	KJ617332.1	
147	Katsuwonus pelamis	KJ617326.1	
148	Katsuwonus pelamis	KJ617325.1	
149	Katsuwonus pelamis	KJ617324.1	
150	Katsuwonus pelamis	KJ617320.1	
151	Katsuwonus pelamis	KJ617318.1	
152	Katsuwonus pelamis	KJ617309.1	
153	Katsuwonus pelamis	KJ617308.1	
154	Katsuwonus pelamis	KJ617306.1	
155	Katsuwonus pelamis	KJ617299.1	
156	Katsuwonus pelamis	KJ617297.1	
157	Katsuwonus pelamis	KJ617283.1	
158	Katsuwonus pelamis	KJ617273.1	
159	Katsuwonus pelamis	KJ617260.1	
160	Katsuwonus pelamis	KJ617259.1	
161	Rastrelliger faughni	LC064498.1	
162	Rastrelliger faughni	LC064497.1	
163	Rastrelliger faughni	LC064496.1	
164	Rastrelliger faughni	LC064495.1	
165	Rastrelliger faughni	LC064494.1	
166	Rastrelliger faughni	LC064493.1	
167	Rastrelliger faughni	LC064490.1	
168	Rastrelliger faughni	LC064489.1	
169	Rastrelliger faughni	LC064488.1	
170	Rastrelliger faughni	LC064487.1	
171	Rastrelliger faughni	LC064486.1	
172	Rastrelliger faughni	LC064485.1	
173	Rastrelliger faughni	LC064484.1	
174	Rastrelliger faughni	LC064483.1	
175	Rastrelliger faughni	LC064482.1	
176	Rastrelliger faughni	LC064481.1	
177	Rastrelliger faughni	LC064480.1	
178	Rastrelliger faughni	LC064479.1	
179	Rastrelliger faughni	LC064492.1	
180	Rastrelliger faughni	LC064491.1	
181	Rastrelliger faughni	LC064478.1	
182	Rastrelliger faughni	LC064477.1	
183	Rastrelliger faughni	LC064476.1	
184	Rastrelliger faughni	LC064475.1	
185	Rastrelliger faughni	DQ497851.1	
186	Rastrelliger faughni	DQ497850.1	
187	Rastrelliger faughni	DQ497849.1	
188	Rastrelliger faughni	DQ497848.1	
189	Rastrelliger faughni	DQ497847.1	
190	Rastrelliger faughni	DQ497845.1	
191	Rastrelliger faughni	DQ497844.1	
192	Rastrelliger kanagurta	JQ681725.1	
193	Rastrelliger kanagurta	JQ681724.1	
194	Rastrelliger kanagurta	JQ681714.1	
195	Rastrelliger kanagurta	JQ681713.1	
196	Rastrelliger kanagurta	JQ681698.1	
197	Rastrelliger kanagurta	JQ681697.1	
198	Rastrelliger kanagurta	JQ681693.1	
199	Rastrelliger kanagurta	JQ681688.1	
200	Rastrelliger kanagurta	JQ681682.1	
201	Rastrelliger kanagurta	JQ681680.1	
202	Rastrelliger kanagurta	JQ681674.1	
203	Rastrelliger kanagurta	JQ681672.1	
204	Rastrelliger kanagurta	JQ681670.1	
205	Rastrelliger kanagurta	JQ681662.1	
206	Rastrelliger kanagurta	JQ681661.1	
207	Rastrelliger kanagurta	JQ681659.1	
208	Rastrelliger kanagurta	JQ681655.1	
209	Rastrelliger kanagurta	JQ681644.1	
210	Rastrelliger kanagurta	JQ681642.1	
211	Rastrelliger kanagurta	JQ681640.1	
212	Rastrelliger kanagurta	JQ681637.1	
213	Rastrelliger kanagurta	JQ681618.1	
214	Rastrelliger kanagurta	JQ681603.1	
215	Rastrelliger kanagurta	JQ681598.1	
216	Rastrelliger kanagurta	JQ681597.1	
217	Rastrelliger kanagurta	JQ681589.1	
218	Rastrelliger kanagurta	JQ681566.1	
219	Rastrelliger kanagurta	JQ681564.1	
220	Rastrelliger kanagurta	JQ681553.1	
221	Rastrelliger kanagurta	JQ681552.1	
222	Rastrelliger kanagurta	KM016914.1	
223	Rastrelliger kanagurta	LC064563.1	
224	Rastrelliger kanagurta	LC064562.1	
225	Rastrelliger kanagurta	LC064561.1	
226	Rastrelliger kanagurta	LC064560.1	
227	Rastrelliger kanagurta	LC064559.1	
228	Rastrelliger kanagurta	LC064558.1	
229	Rastrelliger kanagurta	LC064557.1	
230	Rastrelliger kanagurta	LC064556.1	
231	Rastrelliger kanagurta	LC064555.1	
232	Rastrelliger kanagurta	LC064554.1	
233	Rastrelliger kanagurta	LC064553.1	
234	Rastrelliger kanagurta	LC064552.1	
235	Rastrelliger kanagurta	LC064551.1	
236	Rastrelliger kanagurta	LC064550.1	
237	Rastrelliger kanagurta	LC064549.1	
238	Rastrelliger kanagurta	LC064548.1	
239	Rastrelliger kanagurta	LC064547.1	
240	Rastrelliger kanagurta	LC064546.1	
241	Rastrelliger kanagurta	LC064545.1	
242	Rastrelliger kanagurta	LC064544.1	
243	Rastrelliger kanagurta	LC064543.1	
244	Rastrelliger kanagurta	LC064542.1	
245	Rastrelliger kanagurta	LC064541.1	
246	Rastrelliger kanagurta	LC064540.1	
247	Rastrelliger kanagurta	LC064539.1	
248	Rastrelliger kanagurta	LC064538.1	
249	Rastrelliger kanagurta	LC064537.1	
250	Rastrelliger kanagurta	LC064536.1	
251	Rastrelliger kanagurta	LC064535.1	
252	Rastrelliger kanagurta	LC064534.1	
253	Rastrelliger kanagurta	LC064533.1	
254	Rastrelliger kanagurta	LC064532.1	
255	Rastrelliger kanagurta	LC064531.1	
256	Rastrelliger kanagurta	LC064530.1	
257	Rastrelliger kanagurta	LC064529.1	
258	Rastrelliger kanagurta	LC064528.1	
259	Rastrelliger kanagurta	LC064527.1	
260	Rastrelliger kanagurta	LC064526.1	
261	Rastrelliger kanagurta	LC064525.1	
262	Rastrelliger kanagurta	LC064524.1	
263	Rastrelliger kanagurta	LC064523.1	
264	Rastrelliger kanagurta	LC064522.1	
265	Rastrelliger kanagurta	LC064521.1	
266	Rastrelliger kanagurta	LC064520.1	
267	Rastrelliger kanagurta	LC064519.1	
268	Rastrelliger kanagurta	LC064518.1	
269	Rastrelliger kanagurta	LC064517.1	
270	Rastrelliger kanagurta	LC064516.1	
271	Rastrelliger kanagurta	LC064515.1	
272	Rastrelliger kanagurta	LC064514.1	
273	Rastrelliger kanagurta	LC064513.1	
274	Rastrelliger kanagurta	LC064512.1	
275	Rastrelliger kanagurta	LC064511.1	
276	Rastrelliger kanagurta	LC064510.1	
277	Rastrelliger kanagurta	LC064509.1	
278	Rastrelliger kanagurta	LC064508.1	
279	Rastrelliger kanagurta	LC064507.1	
280	Rastrelliger kanagurta	LC064506.1	
281	Rastrelliger kanagurta	LC064505.1	
282	Rastrelliger kanagurta	LC064504.1	
283	Rastrelliger kanagurta	LC064503.1	
284	Rastrelliger kanagurta	LC064502.1	
285	Rastrelliger kanagurta	LC064501.1	
286	Rastrelliger kanagurta	LC064500.1	
287	Rastrelliger kanagurta	LC064499.1	
288	Rastrelliger kanagurta	DQ497857.1	
289	Rastrelliger kanagurta	HM131887.1	
290	Rastrelliger kanagurta	HM131886.1	
291	Rastrelliger kanagurta	JQ681735.1	
292	Rastrelliger kanagurta	JQ681734.1	
293	Rastrelliger kanagurta	JQ681733.1	
294	Rastrelliger kanagurta	JQ681732.1	
295	Rastrelliger kanagurta	JQ681731.1	
296	Rastrelliger kanagurta	JQ681730.1	
297	Rastrelliger kanagurta	JQ681729.1	
298	Rastrelliger kanagurta	JQ681728.1	
299	Rastrelliger kanagurta	JQ681727.1	
300	Rastrelliger kanagurta	JQ681726.1	
301	Rastrelliger kanagurta	JQ681723.1	
302	Rastrelliger kanagurta	JQ681722.1	
303	Rastrelliger kanagurta	JQ681721.1	
304	Rastrelliger kanagurta	JQ681720.1	
305	Rastrelliger kanagurta	JQ681719.1	
306	Rastrelliger kanagurta	JQ681718.1	
307	Rastrelliger kanagurta	JQ681717.1	
308	Rastrelliger kanagurta	JQ681716.1	
309	Rastrelliger kanagurta	JQ681715.1	
310	Rastrelliger kanagurta	JQ681712.1	
311	Rastrelliger kanagurta	JQ681711.1	
312	Rastrelliger kanagurta	JQ681710.1	
313	Rastrelliger kanagurta	JQ681709.1	
314	Rastrelliger kanagurta	JQ681708.1	
315	Rastrelliger kanagurta	JQ681707.1	
316	Rastrelliger kanagurta	JQ681706.1	
317	Rastrelliger kanagurta	JQ681705.1	
318	Rastrelliger kanagurta	JQ681704.1	
319	Rastrelliger kanagurta	JQ681703.1	
320	Rastrelliger kanagurta	JQ681702.1	
321	Rastrelliger kanagurta	JQ681701.1	
322	Rastrelliger kanagurta	JQ681700.1	
323	Rastrelliger kanagurta	JQ681699.1	
324	Rastrelliger kanagurta	JQ681696.1	
325	Rastrelliger kanagurta	JQ681695.1	
326	Rastrelliger kanagurta	JQ681694.1	
327	Rastrelliger kanagurta	JQ681692.1	
328	Rastrelliger kanagurta	JQ681691.1	
329	Rastrelliger kanagurta	JQ681690.1	
330	Rastrelliger kanagurta	JQ681689.1	
331	Rastrelliger kanagurta	JQ681687.1	
332	Rastrelliger kanagurta	JQ681686.1	
333	Rastrelliger kanagurta	JQ681685.1	
334	Rastrelliger kanagurta	JQ681684.1	
335	Rastrelliger kanagurta	JQ681683.1	
336	Rastrelliger kanagurta	JQ681679.1	
337	Rastrelliger kanagurta	JQ681678.1	
338	Rastrelliger kanagurta	JQ681677.1	
339	Rastrelliger kanagurta	JQ681676.1	
340	Rastrelliger kanagurta	JQ681675.1	
341	Rastrelliger kanagurta	JQ681673.1	
342	Rastrelliger kanagurta	JQ681671.1	
343	Rastrelliger kanagurta	JQ681669.1	
344	Rastrelliger kanagurta	JQ681668.1	
345	Rastrelliger kanagurta	JQ681667.1	
346	Rastrelliger kanagurta	JQ681666.1	
347	Rastrelliger kanagurta	JQ681665.1	
348	Rastrelliger kanagurta	JQ681664.1	
349	Rastrelliger kanagurta	JQ681663.1	
350	Rastrelliger kanagurta	JQ681660.1	
351	Rastrelliger kanagurta	JQ681658.1	
352	Rastrelliger kanagurta	JQ681657.1	
353	Rastrelliger kanagurta	JQ681656.1	
354	Rastrelliger kanagurta	JQ681654.1	
355	Rastrelliger kanagurta	JQ681653.1	
356	Rastrelliger kanagurta	JQ681652.1	
357	Rastrelliger kanagurta	JQ681651.1	
358	Rastrelliger kanagurta	JQ681650.1	
359	Rastrelliger kanagurta	JQ681649.1	
360	Rastrelliger kanagurta	JQ681648.1	
361	Rastrelliger kanagurta	JQ681647.1	
362	Rastrelliger kanagurta	JQ681646.1	
363	Rastrelliger kanagurta	JQ681645.1	
364	Rastrelliger kanagurta	JQ681643.1	
365	Rastrelliger kanagurta	JQ681641.1	
366	Rastrelliger kanagurta	JQ681639.1	
367	Rastrelliger kanagurta	JQ681638.1	
368	Rastrelliger kanagurta	JQ681636.1	
369	Rastrelliger kanagurta	JQ681635.1	
370	Rastrelliger kanagurta	JQ681634.1	
371	Rastrelliger kanagurta	JQ681633.1	
372	Rastrelliger kanagurta	JQ681632.1	
373	Rastrelliger kanagurta	JQ681631.1	
374	Rastrelliger kanagurta	JQ681630.1	
375	Rastrelliger kanagurta	JQ681629.1	
376	Rastrelliger kanagurta	JQ681628.1	
377	Rastrelliger kanagurta	JQ681627.1	
378	Rastrelliger kanagurta	JQ681626.1	
379	Rastrelliger kanagurta	JQ681625.1	
380	Rastrelliger kanagurta	JQ681624.1	
381	Rastrelliger kanagurta	JQ681623.1	
382	Rastrelliger kanagurta	JQ681622.1	
383	Rastrelliger kanagurta	JQ681621.1	
384	Rastrelliger kanagurta	JQ681620.1	
385	Rastrelliger kanagurta	JQ681619.1	
386	Rastrelliger kanagurta	JQ681617.1	
387	Rastrelliger kanagurta	JQ681616.1	
388	Rastrelliger kanagurta	JQ681615.1	
389	Rastrelliger kanagurta	JQ681614.1	
390	Rastrelliger kanagurta	JQ681613.1	
391	Rastrelliger kanagurta	JQ681612.1	
392	Rastrelliger kanagurta	JQ681611.1	
393	Rastrelliger kanagurta	JQ681610.1	
394	Rastrelliger kanagurta	JQ681609.1	
395	Rastrelliger kanagurta	JQ681608.1	
396	Rastrelliger kanagurta	JQ681607.1	
397	Rastrelliger kanagurta	JQ681605.1	
398	Rastrelliger kanagurta	JQ681604.1	
399	Rastrelliger kanagurta	JQ681602.1	
400	Rastrelliger kanagurta	JQ681601.1	
401	Rastrelliger kanagurta	JQ681600.1	
402	Rastrelliger kanagurta	JQ681599.1	
403	Rastrelliger kanagurta	JQ681596.1	
404	Rastrelliger kanagurta	JQ681595.1	
405	Rastrelliger kanagurta	JQ681594.1	
406	Rastrelliger kanagurta	JQ681593.1	
407	Rastrelliger kanagurta	JQ681592.1	
408	Rastrelliger kanagurta	JQ681591.1	
409	Rastrelliger kanagurta	JQ681590.1	
410	Rastrelliger kanagurta	JQ681588.1	
411	Rastrelliger kanagurta	JQ681587.1	
412	Rastrelliger kanagurta	JQ681586.1	
413	Rastrelliger kanagurta	JQ681585.1	
414	Rastrelliger kanagurta	JQ681584.1	
415	Rastrelliger kanagurta	JQ681583.1	
416	Rastrelliger kanagurta	JQ681582.1	
417	Rastrelliger kanagurta	JQ681581.1	
418	Rastrelliger kanagurta	JQ681580.1	
419	Rastrelliger kanagurta	JQ681579.1	
420	Rastrelliger kanagurta	JQ681578.1	
421	Rastrelliger kanagurta	JQ681577.1	
422	Rastrelliger kanagurta	JQ681576.1	
423	Rastrelliger kanagurta	JQ681575.1	
424	Rastrelliger kanagurta	JQ681574.1	
425	Rastrelliger kanagurta	JQ681573.1	
426	Rastrelliger kanagurta	JQ681572.1	
427	Rastrelliger kanagurta	JQ681571.1	
428	Rastrelliger kanagurta	JQ681570.1	
429	Rastrelliger kanagurta	JQ681569.1	
430	Rastrelliger kanagurta	JQ681568.1	
431	Rastrelliger kanagurta	JQ681567.1	
432	Rastrelliger kanagurta	JQ681565.1	
433	Rastrelliger kanagurta	JQ681563.1	
434	Rastrelliger kanagurta	JQ681562.1	
435	Rastrelliger kanagurta	JQ681561.1	
436	Rastrelliger kanagurta	JQ681560.1	
437	Rastrelliger kanagurta	JQ681559.1	
438	Rastrelliger kanagurta	JQ681558.1	
			
439	Rastrelliger kanagurta	JQ681557.1	
440	Rastrelliger kanagurta	JQ681556.1	
441	Rastrelliger kanagurta	JQ681555.1	
442	Rastrelliger kanagurta	JQ681554.1	
443	Rastrelliger kanagurta	JQ681551.1	
444	Rastrelliger kanagurta	JQ681550.1	
445	Rastrelliger kanagurta	JQ681549.1	
446	Rastrelliger kanagurta	JQ681548.1	
447	Rastrelliger kanagurta	JQ681547.1	
448	Rastrelliger kanagurta	JQ681546.1	
449	Rastrelliger kanagurta	JQ681545.1	
450	Rastrelliger kanagurta	JQ681544.1	
451	Rastrelliger kanagurta	JQ681543.1	
452	Rastrelliger kanagurta	JQ681542.1	
453	Rastrelliger kanagurta	DQ497859.1	
454	Rastrelliger kanagurta	DQ497858.1	
455	Rastrelliger_brachysoma	AB507243.1	
456	Rastrelliger_brachysoma	AB507242.1	
457	Rastrelliger_brachysoma	AB507241.1	
458	Rastrelliger_brachysoma	AB507240.1	
459	Rastrelliger_brachysoma	AB507239.1	
460	Rastrelliger_brachysoma	AB507238.1	
461	Rastrelliger_brachysoma	AB507237.1	
462	Rastrelliger_brachysoma	AB507236.1	
463	Rastrelliger_brachysoma	AB507235.1	
464	Rastrelliger_brachysoma	AB507234.1	
465	Rastrelliger_brachysoma	AB507233.1	
466	Rastrelliger_brachysoma	AB507232.1	
467	Rastrelliger_brachysoma	AB507231.1	
468	Rastrelliger_brachysoma	AB507230.1	
469	Rastrelliger_brachysoma	AB507229.1	
470	Rastrelliger_brachysoma	AB507228.1	
471	Rastrelliger_brachysoma	AB507227.1	
472	Rastrelliger_brachysoma	AB507226.1	
473	Rastrelliger_brachysoma	AB507225.1	
474	Rastrelliger_brachysoma	AB507224.1	
475	Rastrelliger_brachysoma	AB507223.1	
476	Rastrelliger_brachysoma	AB507222.1	
477	Rastrelliger_brachysoma	AB507221.1	
478	Rastrelliger_brachysoma	AB507220.1	
479	Rastrelliger_brachysoma	AB507219.1	
480	Rastrelliger_brachysoma	AB507218.1	
481	Rastrelliger_brachysoma	AB507217.1	
482	Rastrelliger_brachysoma	AB507216.1	
483	Rastrelliger_brachysoma	AB507215.1	
484	Rastrelliger_brachysoma	AB507214.1	
485	Rastrelliger_brachysoma	AB507213.1	
486	Rastrelliger_brachysoma	AB507212.1	
487	Scomberomorus commerson	HQ403349.1	
488	Scomberomorus commerson	HQ403348.1	
489	Scomberomorus commerson	HQ403347.1	
490	Scomberomorus commerson	HQ403346.1	
491	Scomberomorus commerson	HQ403345.1	
492	Scomberomorus commerson	HQ403344.1	
493	Scomberomorus commerson	HQ403343.1	
494	Scomberomorus commerson	HQ403342.1	
495	Scomberomorus commerson	HQ403341.1	
496	Scomberomorus commerson	HQ403340.1	
497	Scomberomorus commerson	HQ403339.1	
498	Scomberomorus commerson	HQ403338.1	
499	Scomberomorus commerson	HQ403337.1	
500	Scomberomorus commerson	HQ403336.1	
501	Scomberomorus commerson	HQ403335.1	
502	Scomberomorus commerson	HQ403334.1	
503	Scomberomorus commerson	HQ403333.1	
504	Scomberomorus commerson	HQ403332.1	
505	Scomberomorus commerson	HQ403331.1	
506	Scomberomorus commerson	HQ403330.1	
507	Scomberomorus commerson	HQ403329.1	
508	Scomberomorus commerson	HQ403328.1	
509	Scomberomorus commerson	HQ403327.1	
510	Scomberomorus commerson	HQ403326.1	
511	Scomberomorus commerson	HQ403325.1	
512	Scomberomorus commerson	HQ403324.1	
513	Scomberomorus commerson	HQ403323.1	
514	Scomberomorus commerson	HQ403322.1	
515	Scomberomorus commerson	HQ403321.1	
516	Scomberomorus commerson	HQ403320.1	
517	Scomberomorus commerson	HQ403319.1	
518	Scomberomorus commerson	HQ403318.1	
519	Scomberomorus commerson	HQ403317.1	
520	Scomberomorus commerson	HQ403316.1	
521	Scomberomorus commerson	HQ403315.1	
522	Scomberomorus commerson	HQ403314.1	
523	Scomberomorus commerson	HQ403313.1	
524	Scomberomorus commerson	HQ403312.1	
525	Scomberomorus commerson	HQ403311.1	
526	Scomberomorus commerson	HQ403310.1	
527	Scomberomorus commerson	HQ403309.1	
528	Scomberomorus commerson	HQ403308.1	
529	Scomberomorus commerson	HQ403307.1	
530	Scomberomorus commerson	HQ403306.1	
531	Scomberomorus commerson	HQ403305.1	
532	Scomberomorus commerson	HQ403304.1	
533	Scomberomorus commerson	HQ403303.1	
534	Scomberomorus commerson	HQ403302.1	
535	Scomberomorus commerson	HQ403301.1	
536	Scomberomorus commerson	HQ403300.1	
537	Scomberomorus commerson	HQ403299.1	
538	Scomberomorus commerson	HQ403298.1	
539	Scomberomorus commerson	HQ403297.1	
540	Scomberomorus commerson	HQ403296.1	
541	Scomberomorus commerson	HQ403295.1	
542	Scomberomorus commerson	HQ403294.1	
543	Scomberomorus commerson	HQ403293.1	
544	Scomberomorus commerson	HQ403292.1	
545	Scomberomorus commerson	HQ403291.1	
546	Scomberomorus commerson	HQ403290.1	
547	Scomberomorus commerson	HQ403289.1	
548	Scomberomorus commerson	HQ403288.1	
549	Scomberomorus commerson	HQ403287.1	
550	Scomberomorus commerson	HQ403286.1	
551	Scomberomorus commerson	HQ403285.1	
552	Scomberomorus commerson	HQ403284.1	
553	Scomberomorus commerson	HQ403283.1	
554	Scomberomorus commerson	HQ403282.1	
555	Scomberomorus commerson	HQ403281.1	
556	Scomberomorus commerson	HQ403280.1	
557	Scomberomorus commerson	HQ403279.1	
558	Scomberomorus commerson	HQ403278.1	
559	Scomberomorus commerson	HQ403277.1	
560	Scomberomorus commerson	HQ403276.1	
561	Scomberomorus commerson	HQ403275.1	
562	Scomberomorus commerson	HQ403274.1	
563	Scomberomorus commerson	HQ403273.1	
564	Scomberomorus commerson	HQ403272.1	
565	Scomberomorus commerson	HQ403271.1	
566	Scomberomorus commerson	HQ403270.1	
567	Scomberomorus commerson	HQ403269.1	
568	Scomberomorus commerson	HQ403268.1	
569	Scomberomorus commerson	HQ403267.1	
570	Scomberomorus commerson	HQ403266.1	
571	Scomberomorus commerson	HQ403265.1	
572	Scomberomorus commerson	HQ403264.1	
573	Scomberomorus commerson	HQ403263.1	
574	Scomberomorus commerson	HQ403262.1	
575	Scomberomorus commerson	HQ403261.1	
576	Scomberomorus commerson	HQ403260.1	
577	Scomberomorus commerson	HQ403258.1	
578	Scomberomorus commerson	HQ403257.1	
579	Scomberomorus commerson	HQ403256.1	
580	Scomberomorus commerson	HQ403255.1	
581	Scomberomorus guttatus 	DQ497880.1	
582	Scomberomorus guttatus 	DQ497879.1	
583	Scomberomorus guttatus 	DQ497878.1	
584	Scomberomorus guttatus 	DQ497877.1	
585	Scomberomorus guttatus 	DQ497876.1	
586	Scomberomorus guttatus 	DQ497875.1	
587	Scomberomorus guttatus 	DQ497874.1	
588	Scomberomorus guttatus 	DQ497873.1	
589	Scomberomorus guttatus 	DQ497872.1	
590	Scomberomorus guttatus 	DQ497871.1	
591	Scomberomorus guttatus	DQ497870.1	
592	Scomberomorus guttatus	DQ497869.1	
593	Scomberomorus guttatus	DQ497868.1	
594	Sarda orientalis	EF141178.1	
595	Sarda orientalis	DQ497890.1	
596	Sarda orientalis	DQ497889.1	
597	Sarda orientalis	DQ497888.1	
598	Thunnus alalunga	KR023771.1	
599	Thunnus alalunga	KR023770.1	
600	Thunnus alalunga	KR023769.1	
601	Thunnus alalunga	KR023768.1	
602	Thunnus alalunga	KR023767.1	
603	Thunnus alalunga	KR023766.1	
604	Thunnus alalunga	KR023765.1	
605	Thunnus alalunga	KR023764.1	
606	Thunnus alalunga	KR023763.1	
607	Thunnus alalunga	KR023762.1	
608	Thunnus alalunga	KR023761.1	
609	Thunnus alalunga	KR023760.1	
610	Thunnus alalunga	KR023759.1	
611	Thunnus alalunga	KR023758.1	
612	Thunnus alalunga	KR023757.1	
613	Thunnus alalunga	KR023756.1	
614	Thunnus alalunga	KR023755.1	
615	Thunnus alalunga	KR023754.1	
616	Thunnus alalunga	KR023753.1	
617	Thunnus alalunga	KR023752.1	
618	Thunnus alalunga	KR023751.1	
619	Thunnus alalunga	KR023750.1	
620	Thunnus alalunga	KR023749.1	
621	Thunnus alalunga	KR023748.1	
622	Thunnus alalunga	KR023747.1	
623	Thunnus alalunga	KR023746.1	
624	Thunnus alalunga	KR023745.1	
625	Thunnus alalunga	KR023744.1	
626	Thunnus alalunga	KR023743.1	
627	Thunnus alalunga	KR023742.1	
628	Thunnus alalunga	KR023741.1	
629	Thunnus alalunga	KR023740.1	
630	Thunnus alalunga	KR023739.1	
631	Thunnus alalunga	KR023738.1	
632	Thunnus alalunga	KR023737.1	
633	Thunnus alalunga	KR023736.1	
634	Thunnus alalunga	KR023735.1	
635	Thunnus alalunga	KR023734.1	
636	Thunnus alalunga	EU224087.1	
637	Thunnus alalunga	DQ198012.1	
638	Thunnus alalunga	EF392628.1	
639	Thunnus alalunga	EF392627.1	
640	Thunnus alalunga	EU036521.1	
641	Thunnus alalunga	EU036520.1	
642	Thunnus alalunga	EF439239.1	
643	Thunnus alalunga	EF439238.1	
644	Thunnus alalunga	EF439607.1	
645	Thunnus alalunga	EF439606.1	
646	Thunnus alalunga	EF427610.1	
647	Thunnus alalunga	EF427609.1	
648	Thunnus alalunga	DQ080299.1	
649	Thunnus alalunga	DQ080298.1	
650	Thunnus alalunga	DQ080297.1	
651	Thunnus alalunga	DQ080296.1	
652	Thunnus alalunga	DQ080295.1	
653	Thunnus alalunga	DQ080294.1	
654	Thunnus alalunga	DQ080293.1	
655	Thunnus alalunga	DQ080292.1	
656	Thunnus alalunga	DQ080291.1	
657	Thunnus alalunga	DQ080290.1	
658	Thunnus alalunga	DQ080289.1	
659	Thunnus alalunga	MG017701.1	
660	Thunnus alalunga	MG017700.1	
661	Thunnus alalunga	MG017699.1	
662	Thunnus alalunga	MG017698.1	
663	Thunnus alalunga	EF141184.1	
664	Thunnus alalunga	EF141182.1	
665	Thunnus alalunga	DQ497897.1	
666	Thunnus alalunga	DQ497896.1	
667	Thunnus alalunga	DQ497895.1	
668	Thunnus albacares	KJ531379.1	
669	Thunnus albacares	KJ531378.1	
670	Thunnus albacares	KJ531377.1	
671	Thunnus albacares	KJ531376.1	
672	Thunnus albacares	KJ531375.1	
673	Thunnus albacares	KJ531374.1	
674	Thunnus albacares	KJ531373.1	
675	Thunnus albacares	KJ531372.1	
676	Thunnus albacares	KJ531371.1	
677	Thunnus albacares	KJ531370.1	
678	Thunnus albacares	KJ531369.1	
679	Thunnus albacares	KJ531368.1	
680	Thunnus albacares	KJ531367.1	
681	Thunnus albacares	KJ531366.1	
682	Thunnus albacares	KJ531365.1	
683	Thunnus albacares	KJ531364.1	
684	Thunnus albacares	KJ531363.1	
685	Thunnus albacares	KJ531362.1	
686	Thunnus albacares	KJ531361.1	
687	Thunnus albacares	KJ531359.1	
688	Thunnus albacares	KJ531358.1	
689	Thunnus albacares	KJ531357.1	
690	Thunnus albacares	KJ531356.1	
691	Thunnus albacares	KJ531354.1	
692	Thunnus albacares	KJ531353.1	
693	Thunnus albacares	KJ531352.1	
694	Thunnus albacares	KJ531351.1	
695	Thunnus albacares	KJ531350.1	
696	Thunnus albacares	KJ531349.1	
697	Thunnus albacares	KJ531348.1	
698	Thunnus albacares	KJ531347.1	
699	Thunnus albacares	KJ531346.1	
700	Thunnus albacares	KJ531345.1	
701	Thunnus albacares	KJ531344.1	
702	Thunnus albacares	KJ531343.1	
703	Thunnus albacares	KJ531342.1	
704	Thunnus albacares	KJ531341.1	
705	Thunnus albacares	KJ531340.1	
706	Thunnus albacares	KJ531338.1	
707	Thunnus albacares	KJ531337.1	
708	Thunnus albacares	KJ531334.1	
709	Thunnus albacares	KJ531332.1	
710	Thunnus albacares	KJ531331.1	
711	Thunnus albacares	KJ531330.1	
712	Thunnus albacares	KJ531329.1	
713	Thunnus albacares	KJ531328.1	
714	Thunnus albacares	KJ531325.1	
715	Thunnus albacares	KJ531324.1	
716	Thunnus albacares	KJ531323.1	
717	Thunnus albacares	KJ531322.1	
718	Thunnus albacares	KJ531320.1	
719	Thunnus albacares	KJ531319.1	
720	Thunnus albacares	KJ531318.1	
721	Thunnus albacares	KJ531317.1	
722	Thunnus albacares	KJ531316.1	
723	Thunnus albacares	KJ531313.1	
724	Thunnus tonggol	EF141181.1	
725	Thunnus tonggol	EF141181.1	
726	Thunnus tonggol	DQ497920.1	
727	Thunnus tonggol	DQ497919.1	
728	Thunnus tonggol	DQ497918.1	
729	Thunnus tonggol	DQ497917.1	
730	Thunnus tonggol	DQ497916.1	
731	Thunnus tonggol	DQ497915.1	
732	Thunnus tonggol	DQ497914.1	
733	Thunnus tonggol	DQ497913.1	
734	Thunnus_obesus	KJ018968.1	
735	Thunnus_obesus	KJ018967.1	
736	Thunnus_obesus	KJ018966.1	
737	Thunnus_obesus	KJ018965.1	
738	Thunnus_obesus	KJ018964.1	
739	Thunnus_obesus	KJ018963.1	
740	Thunnus_obesus	KJ018962.1	
741	Thunnus_obesus	KJ018961.1	
742	Thunnus_obesus	KJ018959.1	
743	Thunnus_obesus	KJ018958.1	
744	Thunnus_obesus	KJ018957.1	
745	Thunnus_obesus	KJ018956.1	
746	Thunnus_obesus	KJ018955.1	
747	Thunnus_obesus	KJ018954.1	
748	Thunnus_obesus	KJ018953.1	
749	Thunnus_obesus	KJ018952.1	
750	Thunnus_obesus	KJ018951.1	
751	Thunnus_obesus	KJ018950.1	
752	Thunnus_obesus	KJ018949.1	
753	Thunnus_obesus	KJ018948.1	
754	Thunnus_obesus	KJ018947.1	
755	Thunnus_obesus	KJ018946.1	
756	Thunnus_obesus	KJ018945.1	
757	Thunnus_obesus	KJ018944.1	
758	Thunnus_obesus	KJ018943.1	
759	Thunnus_obesus	KJ018942.1	
760	Thunnus_obesus	KJ018941.1	
761	Thunnus_obesus	KJ018940.1	
762	Thunnus_obesus	KJ018939.1	
763	Thunnus_obesus	KJ018938.1	
764	Thunnus_obesus	KJ018937.1	
765	Thunnus_obesus	KJ018936.1	
766	Thunnus_obesus	KJ018935.1	
767	Thunnus_obesus	KJ018934.1	
768	Thunnus_obesus	KJ018933.1	
769	Thunnus_obesus	KJ018932.1	
770	Thunnus_obesus	KJ018931.1	
771	Thunnus_obesus	KJ018930.1	
772	Thunnus_obesus	KJ018929.1	
773	Thunnus_obesus	KJ018928.1	
774	Thunnus_obesus	KJ018927.1	
775	Thunnus_obesus	KJ018926.1	
776	Thunnus_obesus	KJ018925.1	
